# Investigating the Effectiveness of Current and Modified World Health Organization Guidelines for the Control of Soil-Transmitted Helminth Infections

**DOI:** 10.1093/cid/ciy002

**Published:** 2018-06-01

**Authors:** Sam H Farrell, Luc E Coffeng, James E Truscott, Marleen Werkman, Jaspreet Toor, Sake J de Vlas, Roy M Anderson

**Affiliations:** 1 London Centre for Neglected Tropical Disease Research, Department of Infectious Disease Epidemiology, St Mary’s Campus, Imperial College London, United Kingdom; 2 Department of Public Health, Erasmus MC, University Medical Center Rotterdam, The Netherlands; 3 DeWorm3 Project, Natural History Museum of London, United Kingdom

**Keywords:** soil-transmitted helminths, control, WHO guidelines, mass drug administration, mathematical modelling

## Abstract

**Background:**

Considerable efforts have been made to better understand the effectiveness of large-scale preventive chemotherapy therapy for the control of morbidity caused by infection with soil-transmitted helminths (STHs): *Ascaris lumbricoides*, *Trichuris trichiura*, and the 2 hookworm species, *Necator americanus* and *Ancylostoma duodenale*. Current World Health Organization (WHO) guidelines for STH control include mass drug administration (MDA) programs based on prevalence measurements, aiming at reducing morbidity in pre–school-aged children (pre-SAC) and school-aged children (SAC) by lowering the prevalence of moderate- to heavy-intensity infections to <1%.

**Methods:**

We project the likely impact of following the current WHO guidelines and assess whether the WHO morbidity goals will be achieved across a range of transmission settings. We also investigate modifications that could be made to the current WHO treatment guidelines, and project their potential impacts in achieving morbidity and transmission control.

**Results:**

While the standard guidelines are sufficient at low transmission levels, community-wide treatment (ie, involving pre-SAC, SAC, and adults) is essential if WHO morbidity goals are to be met in moderate- to high-transmission settings. Moreover, removing the recommendation of decreasing the treatment frequency at midline (5–6 years after the start of MDA) further improves the likelihood of achieving morbidity control in SAC.

**Conclusions:**

We meld analyses based on 2 mathematical models of parasite transmission and control by MDA for the dominant STH species, to generate a unified treatment approach applicable across all settings, regardless of which STH infection is most common. We recommend clearly defined changes to the current WHO guidelines.

The London Declaration on Neglected Tropical Diseases (NTDs), launched in 2012, has helped to highlight the importance of a wide range of infections, including the soil-transmitted helminths (STHs), which are endemic in many developing countries [1]. These intestinal nematode parasites, namely the roundworm *Ascaris lumbricoides*, the hookworms *Ancylostoma duodenale* and *Necator americanus*, and the whipworm *Trichuris trichiura* are the cause of considerable morbidity [2, 3]. The Global Burden of Diseases, Injuries, and Risk Factors Study 2016 places the prevalence of infection with STHs overall at 1.5 billion cases globally, with around 800 million cases of ascariasis and well in excess of 400 million each of trichuriasis and hookworm disease [4]. Individuals become infected with *Ascaris* and *Trichuris* when they ingest parasitic eggs released into the environment through the feces of infected individuals. Hookworm eggs mature and hatch in the soil, and become larvae, which penetrate through human skin when individuals walk barefoot in contaminated habitats. The severity of morbidity is correlated to the intensity of infection. Moderate- to high-intensity infections are linked to diarrhea, anemia, malnutrition, and physical and cognitive impairments [5, 6]; *Ascaris* and *Trichuris* are most commonly found in school-aged children (SAC) [7], whereas for hookworm the highest worm burdens are typically found in adults [8].

The World Health Organization (WHO) has provided guidelines on large-scale preventive chemotherapy through programs employing mass drug administration (MDA) of the drugs albendazole or mebendazole. The guidelines aim to reduce morbidity from STH infections in pre–school-aged children (pre-SAC) and SAC by reducing the prevalence of moderate- or heavy-intensity infections to <1% in SAC [9], the thresholds for which are parasite-specific and based on fecal egg counts [1]. The WHO recommends scaling up MDA programs to target at least 75% of SAC, and to include individuals who are at risk for morbidity caused by STH infections (ie, pregnant women in the last 2 semesters of pregnancy, lactating women, or adults in high-risk occupations) [3]. Regular treatment is required as eggs or larvae remaining present in the environment for many months can be the cause of reinfection.

Over the past several years, members of the NTD Modelling Consortium have published papers on mathematical models of the transmission dynamics, control, and elimination of STH [8, 10–18]. In this study, we expand on this work through the application of two mathematical models, which differ in structure, developed by Imperial College London (ICL) and Erasmus Medical Center Rotterdam (EMC). We focus on predicting the extent to which the current WHO guidelines for starting, scaling up, and stopping MDA against STHs succeed in meeting the stated morbidity goals, the circumstances under which they fail, and what changes could be made to the guidelines to increase the likelihood of reaching the WHO-defined morbidity goals.

## METHODS

### General Modeling Approach

Individual-based stochastic transmission models, developed independently by ICL and EMC, were used in this study in which changes in the worm burden of all individuals within a defined human host population were simulated over time and across host age classes. Both models assume a high level of aggregation in worm burdens, that is, many individuals have a few worms, and a few individuals have many worms. Birth and death rates were chosen to generate an age distribution typical of sub-Saharan African countries. The acquisition and death of worms are stochastic, with species-specific mean lifespans. Exposure and contribution of the worms to the infective pool in the human habitat are age specific. Both models assume that the worms are polygamous; the EMC model also simulates a prepatent period and insemination of female worms. Assessment of infection levels is made through a diagnostic submodel describing the Kato-Katz egg counting method. Details of the ICL and EMC model structures have been published previously [12, 13, 14, 15, 19].

We assume treatment with albendazole alone unless otherwise stated. An individual’s treatment results in killing each parasite with a probability of 99% (*Ascaris*), 94% (hookworm), or 60% (*Trichuris*, by default, see also below) in line with measured egg reduction rates [20–23]. Each independent village’s parameters are based on field data, fitted to the age-specific prevalence of infection profiles in a specific region. For each simulation, we evaluated whether a weighted mean prevalence of moderate to heavy infection in SAC 10 years after the start of MDA was <1%.

See the Supplementary Data for further details of parameterization, weighting methodology, and the models’ approaches toward describing worm fecundity. See Supplementary Table 1 for moderate to heavy infection intensity thresholds.

### Current WHO Guidelines

**Figure 1.**
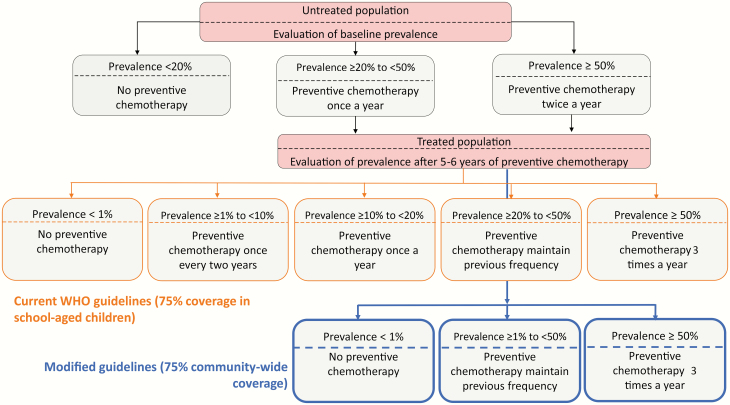
World Health Organization (WHO) decision tree showing the current WHO guidelines to achieve morbidity control in school-aged children (SAC) using 75% coverage in SAC (black and orange boxes). The blue boxes represent the modified guidelines assuming 75% community-wide coverage (pre-SAC, SAC, and adults) to replace the orange boxes (adapted from [1]).

We simulate the impact of the current WHO guidelines (Figure 1), assuming 75% coverage of SAC [1]. Persistent nonadherence was included within the models, which accounts for individuals not taking treatment at any round of MDA [14]. The decision to initiate, continue, or stop MDA within each simulation was based on the measured prevalence in SAC at baseline (before the start for MDA) and midline (5 years after the start of MDA). In both models, prevalences in age groupings (pre-SAC, SAC, and adults, as measured by eggs per gram of feces) vary between simulations due to stochastic effects acting on human and worm demography, and sampling error in diagnostic testing, for which we assume that a single Kato-Katz smear of 41.7 mg is performed [9]. We assume that only SAC are tested to make treatment decisions, even where adults are treated, to allow for continuation of WHO-recommended practices regarding testing programs.

### Modified Guidelines

For simulations where the WHO goal has not been achieved, we explored alternative strategies, such as disregarding the option of reducing the frequency of MDA after the midline evaluation (Figure 1). The options of stopping treatment if the measured prevalence is <1% and increasing treatment frequency at midline are retained. The baseline decisions with regard to MDA frequency were kept the same as in the current WHO guidelines. In addition, the following modifications were considered: (1) MDA targeting SAC at 90% coverage rather than 75%; (2) expanded community-wide MDA at 25%, 50%, or 75% coverage for adults and pre-SAC (in addition to 75% coverage of SAC); and (3) use the drug combination ivermectin and albendazole (rather than albendazole alone) in treating *Trichuris* to increase the effectiveness of treatment. We assumed an increased *Trichuris* worm kill rate per treatment of 95%, when coadministration was applied, instead of 60% [24].

## RESULTS

**Figure 2.**
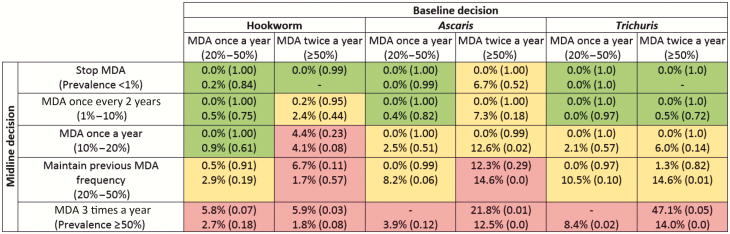
Prevalence of moderate to heavy soil-based helminth infection in school-aged children (SAC) after 10 years of treatment under current guidelines assuming 75% coverage in SAC, highlighted by achievement (green) or otherwise (red) of 1% prevalence goal, or varying predictions between models (yellow), in various transmission settings. Results in each cell have Imperial College London results at the top and Erasmus Medical Center Rotterdam below. Figures in parentheses are the probability of measuring a prevalence of medium- and high-intensity infections <1% from a sample of all SAC. Decisions are based on measured prevalence of infection and correspond with World Health Organization recommendations [1]. Abbreviation: MDA, mass drug administration.

**Figure 3.**
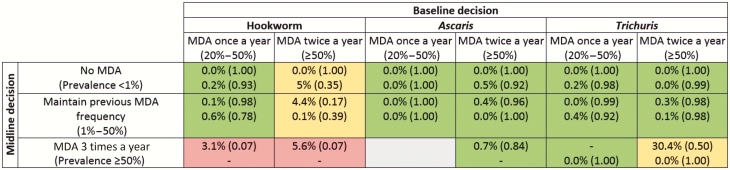
Prevalence of moderate to heavy soil-transmitted helminth infection in school-aged children (SAC) after 10 years of treatment, under recommended updates to treatment guidelines of 75% community-wide coverage. Results in each cell have Imperial College London (ICL) results at the top and Erasmus University Medical Center (EMC) below. Figures in parentheses are the probability of measuring a prevalence of medium- and high-intensity infections <1% from a sample of all SAC. Highlighted by achievement (green) or otherwise (red) of 1% prevalence goal, or varying predictions between models (yellow), in various transmission settings. Results in each cell have ICL results at the top and EMC below. Figures in brackets are the probability of measuring a prevalence of medium- and high-intensity infections <1% from a sample of all SAC. Decisions are based on measured prevalence of infection and correspond with World Health Organization recommendations [1]. The gray box indicates that no villages fell into those categories in either EMC or ICL models. Abbreviation: MDA, mass drug administration.

As infection in each of the simulated villages changes over time, prevalence-based treatment decisions are made per village consistent with the decisions laid out in Figure 1. Ten years after the start of MDA, a simulated Kato-Katz measurement is taken of moderate to heavy infection rates. The mean of villages’ rates in each category of possible decisions is shown in Figures 2 and 3, color-coded by whether the WHO morbidity goal is met.

Figure 2 reveals that for both annual and biannual baseline treatment, the current WHO guidelines are more successful at achieving the morbidity goal if the midline (5–6 years after the start of MDA) medium-heavy prevalence is low. When hookworm and *Trichuris* midline prevalences are <10%, the current WHO guidelines almost always take the prevalence of moderate to heavy infections in SAC to <1%, according to both models. In contrast, both models identify that in scenarios in which prevalence is >50% at baseline and remains so at the midline, twice-yearly followed by thrice-yearly treatment is unable to control transmission for any of the STH species.

In contrast, both models predict a substantial improvement in morbidity outcomes with community-wide treatment, with the WHO morbidity goal being met in most settings (Figure 3). The primary exception is high-transmission hookworm settings, in which outcomes are more mixed. Model predictions differ on whether the goal will be achieved where baseline prevalence exceeds 50%. No villages in the EMC model remained above 50% infection prevalence at the midline evaluation; however, results from the ICL model suggest that those that do (representing areas particularly resilient to treatment) will fail to meet the morbidity target, despite the much broader community-wide coverage.

Figure 2 shows some differences in the predictions of the 2 models to treatment interventions. The descending rows, in each column, show results from villages of increasing prevalence at the midline decision point. Generally, for villages with a low transmission intensity (and so low prevalence at midline), the ICL model shows a lower endline prevalence than the EMC model. For villages with high transmission intensity, the EMC model generally shows the greater response to treatment. These differences reflect the different modeling approaches to impact of individual worm load on egg production. Given identical age-specific levels of infection, adults contribute more to transmission in the EMC model than in the ICL model because of different assumptions about age patterns in the practice of open defecation (see Supplementary Data). As such, the EMC model predicts a larger benefit of community-wide treatment (Figure 3 vs Figure 2) over targeted deworming of SAC compared to the ICL model.

It is important to consider the effect of uncertainty generated by the stochastic nature of the processes being modeled as the endline prevalence results quoted in Figures 2 and 3 are mean values. Prediction intervals for model prevalence estimates are provided in Supplementary Table 2.

The differences between models notwithstanding, our results indicate that current guidelines can produce good outcomes in SAC morbidity control in low-prevalence (and perhaps some moderate-prevalence) areas (Figure 2). However, in higher-prevalence areas, adult infection may be enough to sustain high reinfection of SAC and put achievement of the morbidity goal at risk. In those areas, community-wide transmission is beneficial. In many cases, sufficient community-wide treatment can be expected to break transmission, with obvious benefits for long-term morbidity control.

Both models predict improvement in *Trichuris* outcomes when both albendazole and ivermectin are administered, rather than albendazole only. Applying community-wide treatment has a greater impact on morbidity compared with the drug coadministration strategy (Supplementary Figure 1).

## DISCUSSION

Using two different mathematical models of STH transmission and control, we have investigated the impact of the current WHO guidelines on the likelihood of achieving the STH morbidity goals in SAC. In cases where the morbidity goals are not achieved, we propose modifications of these guidelines as summarized in Table 1.

**Table 1.** Summary of Success or Failure to Meet World Health Organization Morbidity Goal of <1% Prevalence of Moderate- to Heavy-Intensity Infections in School-Aged Children

Current Guidelines	Recommended Updates
There is a high likelihood of failing to achieve the WHO morbidity goals as the baseline prevalence increases. Options where the treatment frequency is reduced at midline are unlikely to reach the morbidity goals. In general, if prevalence of infection remains high (≥20%) at the midline evaluation, models predict that MDA will fail to achieve the morbidity goal. Prevalence of infection <20% at midline indicates achievement of the goal in some circumstances.	We recommend providing 75% community-wide coverage while removing the option to reduce the treatment frequency. In areas where *Trichuris* is dominant, coadministration of albendazole and ivermectin would also be beneficial.

Prevalence of infection refers to measurements in school-aged children. Recommended updates are as follows: 75% community-wide treatment and no reduction in treatment frequency (unless prevalence falls below 1%, treatment may be stopped; follow-up assessments recommended). See also Figure 1 for prevalence-based treatment decisions.

Abbreviations: MDA, drug administration; WHO, World Health Organization.

Current WHO treatment guidelines recommend ending MDA if infection prevalence has been reduced to <1% after 5–6 years of treatment. Our analyses suggest that a 1% prevalence threshold is not always a good criterion on which to judge whether or not to cease treatment. We have accounted for the poor sensitivity of Kato-Katz diagnostics at low prevalence, but poor accessibility of individuals or groups may hide infection from surveillance, something the models do not account for at present.

Updated guidelines for STH MDA have recently been made public [25]. The new guidelines do not include explicit recommendations that treatment frequency be reduced at midline evaluation. This is in line with our findings. In addition, the guidelines make explicit that women of childbearing age should be treated, except those in the first trimester of pregnancy. While a step in the right direction, our findings suggest that coverage short of the entire community at a level of 75% is unlikely to result in achievement of the morbidity goals in SAC, particularly in higher-prevalence areas.

The same guideline updates change the overall prevalence threshold in WHO treatment guidelines for discontinuing MDA from 1% to 2%. Pragmatic concerns regarding the difficulty of measuring prevalence <1% must be taken into account; nevertheless, if such a threshold is to be useful, it must be based on sound evidence. Recent work has shown progress in determining appropriate infection thresholds for the purpose of measuring true interruption of STH transmission [26, 27].

Moreover, our results show that discontinuing MDA on the basis of having achieved even a 1% prevalence threshold may be premature in most high-transmission settings, and ineffective in achieving the stated control aims without careful follow-up evaluation. Though the measured prevalence of infection in SAC may be low, stopping treatment may nevertheless lead to reoccurrence of transmission and “bounce back” to pretreatment levels of infection in part due to the reservoir of infection in adults. We recommend that if MDA is stopped, rigorous surveillance remain in place to prevent morbidity associated with STH infection returning.

A bounce-back of infection after cessation of MDA may provide a window for a subpopulation of parasites that is (partially) treatment resistant on the brink of extinction to reconstitute. To what extent and how fast preventive chemotherapy leads to anthelminthic resistance in STH is uncertain at present, but it is an active area of research. It is of obvious relevance given the observation of widespread drug resistance in helminth species infecting small ruminants and cattle, which are also controlled by means of mass chemoprophylaxis [28, 29]. As in veterinary medicine, development of resistance is probably best delayed by the use of multiple drug combinations and reductions in environmental contamination and/or exposure (ie, improved access to water, hygiene, and sanitation).

The current WHO guidelines do not explicitly recommend follow-up for STH infection detection after cessation of MDA once the morbidity goals have been reached [1]. Parallel WHO guidelines for treatment of schistosomiasis do include a recommendation for serology to be carried out if prevalence <1% in SAC following 5–6 years of treatment [1, 30]. Though there are no commercially available serology tests available for STH, new, more sensitive diagnostic techniques are emerging [31]. We recommend that the new quantitative polymerase chain reaction diagnostic techniques to detect parasite DNA in feces be applied in some trial settings to help ascertain if low-intensity infections persist after MDA cessation. Serology tests may still give a positive outcome after the infection is cleared, as antibodies remain present for some time post-clearance (if not lifelong).

Given the potential for a reservoir of infection in adults if treatment remains SAC focused, surveillance of the entire population across all age groups via transmission assessment surveys is to be recommended. Hence, a key part of deciding whether community-wide MDA is appropriate should be an assessment of the level of infection in adults.

As parasite elimination is approached, issues related to reaching the last few infected individuals come to the fore. This is an area of active research. Recent work on systematic noncompliance with MDA treatment indicates that the importance of this factor to effective control by MDA varies between helminth species, with the longer-lived species perhaps most affected [14, 32]. The importance of accurate coverage data in informing policy is well understood [33]. However, surprisingly, few longitudinal studies on compliance of individuals with anthelmintic treatment have been conducted. Such studies require individuals to be followed up over successive rounds of treatment, over a number of years. This poses considerable logistical challenges. The need for good MDA coverage data has driven the inclusion of individual-level and longitudinal data collection in the DeWorm3 study [34, 35]. Though the 3 dominant STH species are treated collectively in terms of the drug used and the formulation of the WHO treatment guidelines, differences are evident in required action to meet the WHO prevalence goal. Though we mirror current WHO guidelines in producing recommendations that are applicable across STH diseases as a whole, ideally the guidelines would reflect the locally dominant species and provide species-specific recommendations, as well as differentiating by disease prevalence. In very low-prevalence areas (<10%), current guidelines may be sufficient. Less reliably, given the models’ mixed predictions, the goal may be achievable in areas largely free of hookworm with *Ascaris* or *Trichuris* prevalence up to 20%. In all other circumstances, we strongly recommend community-wide treatment at high coverage. Regional circumstances and availability of data on prevalence of the different diseases will determine whether treating STH collectively or tailored by disease and local prevalence is the best approach. Where granular data are not available, we recommend community-wide treatment. In addition, given the low drug efficacy of mebendazole (rather than albendazole) [20–23], we would expect more aggressive, higher-coverage MDA to be necessary where mebendazole is used. In clearing *Trichuris*, a more specific set of guidelines may be needed given the higher efficacy of an albendazole + ivermectin drug coadministration regimen.

We have ensured that our models take account of geographical heterogeneity in transmission by systematically studying a range of parameter values, but have deliberately avoided replicating transmission patterns in any particular region or country. A number of factors will influence transmission patterns within and between specific localities, notably migration of infected individuals between villages or towns, perhaps seasonally for work. This is not accounted for in the current versions of our models, in part because detailed data on such movements are limited to date. However, the availability of relevant data, based on mobile phone usage, is growing in many regions with endemic STH infection (eg, Kenya). Some published research has investigated the impact of human movement [26], but much remains to be explored.

Global efforts to eliminate the NTD lymphatic filariasis as a public health problem [36] provide an opportunity; the drug regimen used in MDA against lymphatic filariasis (LF)—a combination of albendazole and either ivermectin or diethylcarbamazine, community-wide—is also effective against STH. As such, large-scale LF elimination efforts have greatly increased the number of people being treated for STH. As regional LF elimination programs move into transmission assessment surveys to examine suitability for ending MDA, there is scope to coordinate LF surveillance with continued treatment for STH [35]. Missing this opportunity, or treating only children, risks recrudescence and the loss of gains already made against STH infection.

Expanded MDA programs can be considerably more cost-effective than treating smaller subpopulations when economies of scale and the low costs of the (often donated) relevant drugs are taken into account [8, 37]. Coordination between multiple interventions, such as with LF elimination programs, provides scope to reduce costs further.

## CONCLUSIONS

Both modeling approaches agree that in most transmission settings, the present guidelines for treatment coverage need to be broadened to include the treatment of all age groups, to achieve the WHO morbidity goals. Therefore, we recommend attempting to achieve 75% community-wide coverage, while removing the option of reducing the treatment frequency as prevalence drops during a long-term MDA program.

Though WHO goals on STH are focused on morbidity control rather than (as yet) elimination as such, there is obvious benefit to coordinated assessment and continued treatment to achieve more effective morbidity control, and perhaps mitigation of ongoing costs through interruption of parasite transmission.

## Supplementary Data

Supplementary materials are available at *Clinical Infectious Diseases* online. Consisting of data provided by the authors to benefit the reader, the posted materials are not copyedited and are the sole responsibility of the authors, so questions or comments should be addressed to the corresponding author.

Supplementary Table 1

Supplementary Table 2

Supplementary Fig 1

Supplementary Information

## Notes

***Acknowledgments.*** We thank Deirdre Hollingsworth for helpful comments and discussions on the study. Additionally, we thank Hajnal Farkas for managing the NTD Modelling Consortium.

***Disclaimer.*** The views, opinions, assumptions, or any other information set out in this article are solely those of the authors and should not be attributed to the funders or any person connected with the funders. GlaxoSmithKline (R. M. A.) played no role in study design, data collection and analysis, decision to publish, or preparation of the manuscript.

***Financial support.*** This work was supported by the Bill & Melinda Gates Foundation in partnership with the Task Force for Global Health through the NTD Modelling Consortium (award number OPP1053230) and the Children’s Investment Fund Foundation (UK). J. E. T., M. W., and R. M. A. gratefully thank the Bill & Melinda Gates Foundation for research grant support via the DeWorm3 (grant number OPP1129535) award to the Natural History Museum in London. L. E. C. acknowledges funding from the Dutch Society for Scientific Research (NWO/ZonMw, Veni Grant 016.178.156).

***Supplement sponsorship.*** This article appears as part of the supplement “Reaching the 2020 Goals for Nine Neglected Tropical Diseases,” sponsored by the NTD Modelling Consortium.

***Potential conflicts of interest.*** R. M. A. is a nonexecutive director of GlaxoSmithKline. All other authors report no potential conflicts. All authors have submitted the ICMJE Form for Disclosure of Potential Conflicts of Interest. Conflicts that the editors consider relevant to the content of the manuscript have been disclosed.
