# Supplementary material for: Investigating the Effectiveness of Current and Modified World Health Organization Guidelines for the Control of Soil-Transmitted Helminth Infections
Source: Clin Infect Dis. 2018 Jun 1;66(Suppl 4):S253–9. doi: 10.1093/cid/ciy002 (PMC5982801; doi:10.1093/cid/ciy002)
Supplement: Supplementary Table 1 [file ciy002_suppl_supplementary_table_1.docx]

| 1. ***Lumbricoides*** | ***T. trichiura*** | **Hookworms** |
| --- | --- | --- |
| ≥ 5000 e.p.g. | ≥ 1000 e.p.g. | ≥ 2000 e.p.g. |
