# Supplementary material for: Investigating the Effectiveness of Current and Modified World Health Organization Guidelines for the Control of Soil-Transmitted Helminth Infections
Source: Clin Infect Dis. 2018 Jun 1;66(Suppl 4):S253–9. doi: 10.1093/cid/ciy002 (PMC5982801; doi:10.1093/cid/ciy002)
Supplement: Supplementary Table 2 [file ciy002_suppl_supplementary_table_2.docx]

|  | **Hookworm** | | ***Ascaris*** | | ***Trichuris*** | |
| --- | --- | --- | --- | --- | --- | --- |
|  | **MDA once a year** | **MDA twice a year** | **MDA once a year** | **MDA twice a year** | **MDA once a year** | **MDA twice a year** |
| **Any** | NA | NA | NA | NA | NA | NA |
|  | 1.97% (0, 5.08) | 1.71% (0, 5.66) | 3.51% (0, 11.11) | 13.72% (5.71, 21.15) | 7.67% (0, 19.61) | 14.26% (8.06, 24.59) |
| **Stop MDA** | 0.00% (0, 0) | 0% (0, 0) | 0.01% (0, 0) | 0.00% (0, 0) | 0.00% (0, 0) | 0.00% (0, 0) |
|  | 0.24% (0, 0.9) | NA | 0.04% (0, 0) | 4.44% (0, 17.02) | 0.00% (0, 0) | NA |
| **Biennial MDA** | 0.00% (0, 0) | 0% (0, 0) | 0.00% (0, 0) | 0.01% (0, 0) | 0.00% (0, 0) | 0.00% (0, 0) |
|  | 0.5% (0, 1.7) | 2.41% (0, 5.57) | 0.46% (0, 1.56) | 7.38% (0, 15.38) | 0.06% (0, 0) | 0.67% (0, 2.22) |
| **Annual MDA** | 0.00% (0, 0) | 0% (0, 0) | 0.00% (0, 0) | 0.04% (0, 0) | 0.00% (0, 0) | 0.00% (0, 0) |
|  | 0.84% (0, 2.82) | 4.44% (1.43, 6.35) | 2.31% (0, 6.78) | 12.29% (4.95, 20.51) | 1.88% (0, 5.66) | 5.97% (0, 12.13) |
| **Continue initial MDA** | 0.48% (0, 0.64) | 0.48% (0, 0.64) | 0.02% (0, 0) | 12.33% (0, 25.68) | 0.05% (0, 0) | 1.3% (0, 2.72) |
|  | 2.92% (0, 6.17) | 1.64% (0, 5.48) | 8.38% (1.69, 16.47) | 14.64% (7.69, 21.83) | 10.82% (0, 21.65) | 14.68% (5.26, 24.62) |
| **Four-monthly MDA** | 5.76% (2.58, 8.39) | 5.76% (2.58, 8.39) | NA | 21.76% (10.12, 33.55) | NA | 47.06% (8.18, 83.85) |
|  | 2.96% (0, 5.45) | 1.93% (1.72, 2.94) | 4.17% (1.07, 7.05) | 12.82% (6, 19.89) | 8.58% (3.39, 17.45) | 14.1% (8.57, 24.65) |
