# Supplementary figures and images for: Investigating the Effectiveness of Current and Modified World Health Organization Guidelines for the Control of Soil-Transmitted Helminth Infections
Source: Clin Infect Dis. 2018 Jun 1;66(Suppl 4):S253–9. doi: 10.1093/cid/ciy002 (PMC5982801; doi:10.1093/cid/ciy002)

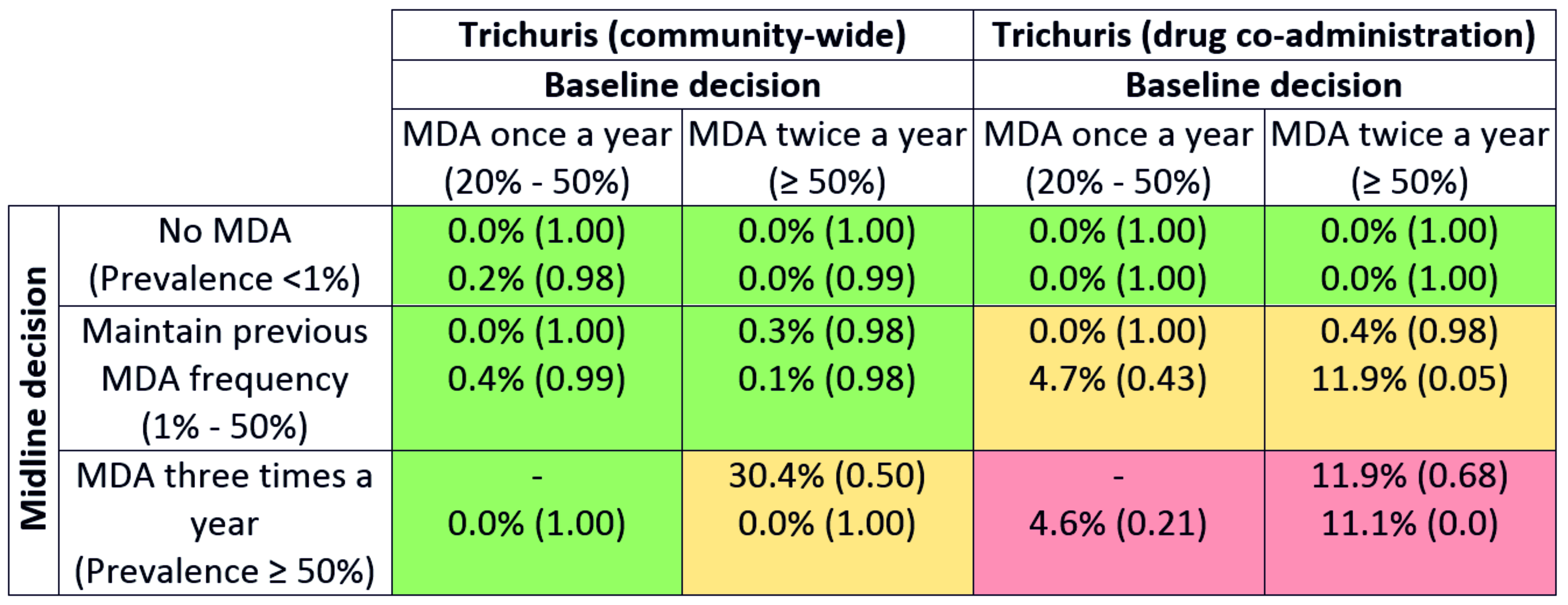

Supplement: Supplementary Fig 1 [file ciy002_suppl_supplementary_fig_1.jpeg]
