# Supplementary material for: Investigating the Effectiveness of Current and Modified World Health Organization Guidelines for the Control of Soil-Transmitted Helminth Infections
Source: Clin Infect Dis. 2018 Jun 1;66(Suppl 4):S253–9. doi: 10.1093/cid/ciy002 (PMC5982801; doi:10.1093/cid/ciy002)
Supplement: Supplementary Information [file ciy002_suppl_supplementary_information.docx]

**Supplementary Information**

Model parameterisation

In the ICL model, each independent village’s parameters are a sample from the likelihood distribution when fitting to real-world data, providing a range of parameter sets. See [1] for a full listing of parameters, including maximum likelihood parameters and details of data. The basic reproductive number R_0_ parameter is randomised. In this manner, a wide range of initial prevalences is arrived at. In the EMC model, the parameter reflecting transmission intensity was varied to obtain a wide range of initial prevalences (other biological parameters were fixed in value). For each STH species, infection in 9,000 (ICL) or 10,000 (EMC) villages were simulated. In both models each village consisted of 500 people with a prescribed age distribution.

Worm fecundity: differences between models

At high host worm burdens, egg production per worm is restricted by overcrowding effects (density dependent fecundity), as recorded in field epidemiological studies involving worm expulsion and faecal egg sampling. In the EMC description, egg production gradually levels off to a maximum level with increasing worm burden. In the ICL description, overcrowding effects lead to the maximum egg production rate being achieved earlier and a subsequent small drop in production for higher worm burdens.

Calculation of weighted average

We summarised the predicted trend in prevalence of moderate to heavy infection per pre-control endemicity category by taking the average over repeated simulations. Because the true distribution of pre-control prevalences in the field is unknown, and because prevalence depends non-linearly on certain model parameters (such as the degree of parasite aggregation), we weighted the individual simulation runs within each category such that their weighted average represented the average of a uniform distribution This weighted average of moderate to heavy infection in each prevalence category was compared to the WHO goal of reduction to less than 1%.

Differences between ICL and EMC models in age-dependent contributions to infection

The differences in predictions for *Ascaris* between models reflect different assumptions about the relative contribution of different age groups to transmission (i.e. the relative frequency of practicing open defaecation; the models employ very similar assumptions about exposure to the environmental reservoir, leading to nearly identical predictions for age profiles in infection levels [2]). Based on the age pattern in hookworm infection levels, the EMC model assumes that the practice of defaecation increases with age up to age ten, and this pattern in open defaecation is then also applied to *Trichuris* and *Ascaris*. In contrast, the ICL model assumes that age-dependent contribution is proportional to age-dependent exposure (i.e. and therefore differs between the three worm species). As such, given identical infection levels in by age, in the EMC model adults contribute relatively more to transmission than in the ICL model, reducing the effectiveness of SAC-targeted MDA, but making community-wide treatment more beneficial.

**References**

1. Truscott JE, Turner HC, Farrell SH, Anderson RM. Soil-transmitted helminths: mathematical models of transmission, the impact of mass drug administration and transmission elimination criteria. Adv Parasitol **2016**; 94: 133-98.

2. Coffeng LE, Truscott JE, Farrell SH, et al. Comparison and validation of two mathematical models for the impact of mass drug administration on *Ascaris lumbricoides* and hookworm infection. Epidemics **2017**; 18: 38-47.
